# NUPR1 contributes to endocrine therapy resistance by modulating BIRC5 expression and inducing luminal B-ERBB2^+^ subtype-like characteristics in estrogen receptor-positive breast cancer cells

**DOI:** 10.7150/jca.105425

**Published:** 2025-02-11

**Authors:** Chun-Hui Lee, Yi-Chen Lin, Yung-Chieh Chang, Pin-Chen Chen, Kai-Hsuan Lin, Tzu-Miao Yeh, Euphemia Yee Leung, I-Li Lin, Shang-Hung Chen, Chun Hei Antonio Cheung

**Affiliations:** 1 Institute of Clinical Medicine, College of Medicine, National Cheng Kung University, Tainan, Taiwan.; 2 Department of Oncology, National Cheng Kung University Hospital, College of Medicine, National Cheng Kung University, Tainan, Taiwan.; 3 Department of Pharmacology, College of Medicine, National Cheng Kung University, Tainan, Taiwan.; 4 TMU Research Center of Cancer Translational Medicine and Taipei Cancer Center, Taipei Medical University Hospital, College of Medicine, Taipei Medical University, Taipei, Taiwan.; 5 National Institute of Cancer Research, National Health Research Institutes, Tainan, Taiwan.; 6 Institute of Basic Medical Sciences, College of Medicine, National Cheng Kung University, Tainan, Taiwan.; 7 Auckland Cancer Society Research Centre and Department of Molecular Medicine and Pathology, University of Auckland, Auckland, New Zealand.; 8 Department of Radiology, Ditmanson Medical Foundation Chia-Yi Christian Hospital, Chiayi, Taiwan.

**Keywords:** Breast cancer, BIRC5, drug resistance, ERBB2, HDAC5, NUPR1

## Abstract

Acquired resistance to endocrine therapy is a major clinical challenge in the treatment of luminal A [estrogen receptor (ER)^+^ and/or progesterone receptor (PR)^+^, human epidermal growth factor receptor 2 (ERBB2/HER2)^-^, and low Ki-67] breast cancer. Recently, molecular subtype conversion has been suggested as one of the possible causes of the development of drug-resistant breast cancer. However, the molecular mechanism underlying the molecular subtype conversion and the induction of endocrine therapy resistance in luminal A breast cancer is still incompletely understood. Here, we found that the ER^+^ MCF7-derived endocrine therapy-resistant MCF7-TamC3 breast cancer cells exhibit increased expression of an intrinsically disordered chromatin protein, NUPR1, compared to the parental luminal-A subtype like MCF7 breast cancer cells. Intriguingly, MCF7-TamC3 cells also exhibit characteristics that resemble the luminal B-ERBB2^+^ breast tumor subtype, like the increased expression of ERBB2 and the increased sensitivity to monoclonal ERBB2-targeting antibody Trastuzumab *in vitro*. Kaplan-Meier analysis of expression cohorts of breast tumors showed that high *NUPR1* mRNA expression levels correlate with poor overall and relapse-free survival in both endocrine therapy-treated ER^+^ and ERBB2-enriched breast cancer patients. Results of the bioinformatics analysis showed that the *NUPR1* mRNA expression level is also correlated with the clinical grading of the Tamoxifen-treated ER^+^ primary breast cancer. The qPCR and the western blot analysis results revealed that NUPR1 positively regulates the expression of the epigenetic regulator HDAC5, the anti-apoptotic molecule BIRC5, and the mitogenic receptor ERBB2 in MCF7-TamC3 and the ERBB2-enriched subtype like SK-BR-3 breast cancer cells. Downregulation of NUPR1 increased the sensitivity to estrogen deprivation in MCF7-TamC3 cells and decreased the viability of SK-BR-3 cells *in vitro*. These findings indicate that dysregulation of NUPR1 promotes the development of estrogen independence in ER^+^ breast cancer cells in part through expression regulation of HDAC5, ERBB2, and BIRC5. Targeting NUPR1 or its downstream regulating molecules may offer a potential strategy for overcoming resistance to endocrine therapy in patients with ER^+^ breast cancer.

## Introduction

Breast cancer is the most common type of cancer among women worldwide. Generally, breast cancer can be classified into four different molecular subtypes, according to the expression/expression levels of estrogen receptor (ER), progesterone receptor (PR), Erb-B2 receptor tyrosine kinase 2 [ERBB2/human epidermal growth factor receptor 2 (HER2)], and Ki-67 (a cell proliferation marker) in cancer cells. Among these breast cancer subtypes [luminal A, luminal B, ERBB2/HER2-positive, and triple-negative (TNBC) breast cancer], luminal A (ER^+^ and/or PR^+^, ERBB2^-^, low Ki-67) is the most common type, which accounts for approximately 40% of all breast cancer cases. For treating breast cancer, therapeutic decisions are mostly made based on cancer subtype classifications. Typically, patients with luminal A breast cancer are treated with endocrine therapies, such as selective estrogen receptor modulators (SERMs; to interfere with the interaction between estrogen and ER) and aromatase inhibitors (AIs; to inhibit estrogen production), whereas anti-ERBB2 treatment can be applied to patients with ERBB2/HER2-positive breast cancer. Despite luminal A breast cancer patients usually showing good initial clinical response to endocrine therapy, resistance to SERMs (*e.g.*, tamoxifen) and AIs (*e.g.*, anastrozole, letrozole, exemestane) is frequently observed, especially in those with prolonged treatments 1, 2. However, the molecular mechanism underlying the induction of endocrine therapy resistance in ER^+^ breast cancer is still incompletely understood.

Emerging evidence shows that molecular subtype conversion frequently occurs during the development of metastatic or drug-resistant breast cancer 3-5. In addition, heterogeneity within molecular subtypes of breast cancer (*i.e.*, intratumoral heterogeneity) is a common phenomenon, and it has been suggested that intratumoral heterogeneity is one of the causes of breast cancer treatment failure 6, 7. Interestingly, recent evidence shows that interconversion between different subtypes of breast cancer cells within a tumor promotes tumor progression and induces treatment resistance 3, 8, 9. The human breast MCF7 cancer cells are commonly used for studying the biology of luminal A breast cancer. This breast cancer cell line was initially thought to be a monoclonal cell line but is now known as a population of breast cancer cells with high levels of molecular heterogeneity (but mostly ER^+^, estrogen-dependent, and SERMs-sensitive) 10, 11. Given the heterogeneous property, this cell line is widely used as a model to study the endocrine therapy/drug resistance development process in ER^+^/luminal A breast cancer by identifying and understanding the molecular profile of the possible drug-resistant subclones (or subpopulations) 12-14.

In a pilot study, we found that the MCF7 cell line-derived, ER^+^, estrogen-independent, and tamoxifen-resistant, MCF7-TamC3 breast cancer cells exhibit increased expression of two epigenetic regulators, HDAC5 and HDAC2, compared to the parental ER^+^ MCF7 breast cancer cells 15. In this study, through cellular and molecular analyses (including transcriptome analysis and bioinformatics analysis, *etc*.) on MCF7 and MCF7-TamC3 breast cancer cells, we further found that MCF7-TamC3 cells exhibit characteristics that resemble the “luminal B-ERBB2 positive” (*i.e.*, ER^+^, ERBB2^+^, and ERBB2-targeting agent-sensitive) breast tumor subtype. Notably, the current study reveals a new role of Nuclear Protein 1 (NUPR1/P8/COM1) in regulating the expression of HDAC5, ERBB2 (and the survival reliance switch from estrogen to EGF) and BIRC5 (a well-known oncogene) in breast cancer cells.

## Methods

### Cell lines and cell culture conditions

The human ER^+^, estrogen-dependent, endocrine therapy sensitive, luminal-A subtype-like MCF7 breast cancer cells were cultured in α-MEM containing 5% FBS, PSG, and insulin-transferring-selenium supplement (ITS) (Diagnostics, cat# 11074547001) 16. The human ER^+^, estrogen-independent, tamoxifen-resistant MCF7-TamC3 cells were obtained from Dr. Euphemia Yee Leung of The University of Auckland, New Zealand, and the cells were created by the prolonged culture of MCF7 cells under estrogen-deprived conditions (to mimic the condition of aromatase inhibitions). The cellular and molecular phenotypes of MCF7-TamC3 breast cancer cells have been characterized partly in previous studies 15, 17. MCF7-TamC3 cells were cultured in phenol-red-free RPMI containing 5% charcoal-stripped FBS, PSG, and ITS. The human ERBB2^+^ (HER2^+^), ERBB2-enriched subtype-like, SK-BR-3 breast cancer cells were originally obtained from ATCC and were cultured in DMEM containing 10% FBS with PSG 16. All the cells were cultured in an incubator containing 5% CO_2_ under 37 ºC. The use of MCF7, MCF7-TamC3, and SK-BR-3 cells in the present study was approved by the biosafety committee of National Cheng Kung University.

### Transcriptome analysis (mRNAseq)

The preparation of all RNA samples was carried out according to Illumina's standard protocol. Briefly, Agilent's SureSelect Strand-Specific RNA Library Preparation Kit was used for library construction, followed by AMPure XP beads (Beckman Coulter, USA) size selection. The sequence was determined using Illumina's sequencing-by-synthesis (SBS) technology for 150 bp paired-end reads. Welgene Biotech's pipeline, based on Illumina's base calling program bcl2fastq (ver. 2.20), was used to generate the sequencing data (FASTQ reads).

Illumina's official tool, bcl2fastq2 conversion Software (ver. 2.19), was used to convert base calling files from all Illumina sequencing systems. Both adaptor clipping and sequence quality trimming were accomplished using the software Trimmomatic (ver. 0.32). HISAT2 was used for mapping next-generation sequencing reads to genomes 18. Differential expression analysis was based on Cuffdiff (@cufflinks 2.2.1) with genome bias detection/correction and Welgene in-house programs 19. Differentially expressed genes (DEGs) of each experiment design were subjected to the enrichment test for functional assay by clusterProfiler (ver. 3.5) 20. A total of 58,229 genes were scanned. DEGs were selected with thresholds of fold change > 2 and *p-value* <0.05. The threshold of > 0.3 FPKM and *q*-value <0.05 [Characteristics of the *p*-value distribution by Benjamini-Hochberg procedure (BH step-up procedure)] for the top 10 DEGs of RNA-seq was determined since this yielded a balance in numbers of false positive and false negative detection and higher confidence in measured expression level.

### Reverse transcription and quantitative real-time polymerase chain reaction (qPCR)

Total RNA was extracted using TRIzol® reagent (Invitrogen, cat# 15596-026), and cDNA was synthesized from total RNA (2 μg) using the RevertAid H Minus First strand cDNA synthesis Kit (Thermo Scientific, cat# K1632). Quantitative real-time PCR was used to determine the relative mRNA expression levels of *NUPR1*, *HDAC5*, *ERBB2*, and *BIRC5* in treated cells using the StepOnePlus™ PCR system. The target fragment was amplified using specific primers [*NUPR1* (Forward primer 5'-GGTCGCACCAAGAGAGAAGC-3'; Reverse primer 5'-CTCCGCAGTCC CATCTCTAT-3'); *HDAC5* (Forward primer 5'-CGCAAGGATGGGACTGTTAT-3'; Reverse primer 5'-GAGCATCTCAGTGGGGATGT-3'); *ERBB2* (Forward primer 5'-CCATAACACCCACCTCTGCT-3'; Reverse primer 5'-ACTGGCTGCAGTTGACACAC-3'); *BIRC5* (Forward primer 5'-AGAACTGGCCCTTCTTGGAGG-3'; Reverse primer 5'-CTTTTTATGTTCCTCTATGGGGTC-3')] and the Fast SYBR® Green Master Mix (Applied Biosystems, cat# 4385612) according to the following protocol: preheating at 95 °C for 20 s, 45 cycles at 95 °C for 1 s and 60 °C for 30 s, and then a dissociation curve performed at 95 °C for 15 s, 60 °C for 60 s, and 95 °C for 15 s. The target genes were quantified using the comparative threshold cycle (Ct) values 2^-ΔΔCt^ method (⊿Ct = Ct_Target_ gene -Ct_Actin_, ⊿⊿Ct = ⊿Ct_Treatment_ -⊿Ct_Control_). Experiments were repeated at least thrice.

### Cell fractionation and nucleic/cytoplasmic protein isolation

Cells were lysed in fraction buffer I containing 1 mM PMSF (Sigma-Aldrich, cat# p7626) and 1 mM NaF with protease inhibitors (Roche) for 15 min on ice. 10% IGEPAL® CA-630 (Sigma-Aldrich, cat# I3021) was added for solubilization, isolation, and purification of membrane protein complexes. After centrifugation, the supernatant (cytoplasmic proteins) and pellet (nucleic proteins) were isolated. Nucleic proteins were extracted by adding fraction buffer II. The protein concentration was measured using bovine serum albumin as standard. Equal amounts of protein were subjected to 12 % SDS-PAGE, and western blot analysis was performed with indicated antibodies.

### Western blot analysis

Cells were lysed using CelLytic™ cell lysis Reagent (Sigma-Aldrich, C2978) containing 1 mM PMSF and 1 mM NaF with cocktail protease inhibitors (Roche, 04693159001) and phosphatase inhibitors (G-Biosciences, 1786-450). Equal amounts of protein were subjected to SDS-PAGE. The resolved proteins were transferred onto a PVDF membrane, which was then exposed to 5% non-fat dried milk/BSA in TBS-Tween buffer for an hour at room temperature before incubation overnight at 4 °C with primary antibodies [anti-ACTA1 (Millipore, cat# MAB1501); anti-BIRC5 (R&D Systems, cat# AF886); anti-CDKN1A (GeneTex, cat# GTX27960); anti-ERBB2 (UltraMAB, cat# UM570036); anti-phospho Tyr1248 ERBB2 (GeneTex, cat# GTX133439); anti-HDAC5 (Proteintech, cat# 16166-1-AP); anti-LMNA (GeneTex, cat# GTX101127); anti-NUPR1 (ABclonal, cat# A18150); anti-TP53 (GeneTex, cat# GTX102965)]. Then, the PVDF membranes were washed thrice with TBS containing 0.05% Tween-20 before incubation for an hour at room temperature with HRP-conjugated secondary antibodies. Immune complexes were detected with chemiluminescence reagents. The luminescence protein signals were detected by Luminescence Readers (FUJI LAS-100, Tokyo, Japan). Experiments were repeated at least thrice.

### MTT cell viability assay

Cells were seeded onto each well of 96-well plates for 24 h before treatment. After treatment, 180 µL of MTT solution (mixing 5 mg/mL MTT solution in phenol-red free RPMI in a ratio of 1:10) was added to each well and incubated for 4 h. Then, 100 µL MTT lysis buffer was added to each well and incubated for 16 h. Cell viability was quantified by measuring the absorbance of the solution at 570 nm wavelength by a spectrophotometer. The percentage of viable cells for each treatment group was calculated by adjusting the untreated control group to 100 %. Triplicate wells were assayed for each condition. Experiments were repeated at least thrice.

### Gene silencing by siRNA

Target-validated siRNA oligomers were transfected into MCF7, MCF7-TamC3, and SK-BR-3 breast cancer cells using Lipofectamine^®^ RNAiMAX reagent (Invitrogen, cat# 13778-150). Cells were seeded onto 96-well plates, 6 cm or 10 cm dishes, and cultured overnight in an antibiotic-free medium. Either the scramble siRNA (Horizon Discovery, cat# D-001206-13-05) or the *NUPR1*-specific siRNA (Horizon Discovery, cat# M-012819-02-0005) oligomers were diluted in Opti-MEM^®^ I medium (Gibco, cat# 31985) without serum, and then mixed with Lipofectamine® RNAiMAX transfection reagent, which was also diluted in Opti-MEM® I medium, for 20 min at room temperature. Cells were overlaid with the transfection mixture and incubated for various durations. Experiments were repeated at least thrice.

### Cell migration analysis (wound healing assay)

A total of 4 x 10^4^ cells were seeded onto each well of the culture inserts (Ibidi, Germany) for 24 h. The culture inserts were removed. Then, 500 μm cell-free gaps were created, and images of the wound areas were taken using an inverted microscope (Nikon E400, Japan) before and after 24 h of treatments. The average width of the wound was measured and analyzed using ImageJ software (National Institutes of Health, USA), and the migratory ability was calculated. Experiments were repeated at least thrice.

### Bioinformatics analysis

The mRNA levels of *NUPR1* in breast cancer with different grades or ERBB2 status were examined through analysis using a database available on Oncomine (www.oncomine.org), which is an open online cancer microarray database to facilitate the discovery of novel biomarkers and therapeutic targets 21. The expression correlation between the mRNA levels of *NUPR1* and the protein levels of ERBB2 in breast cancer cells of different molecular subtypes was also analyzed using a database available on DepMap (https://depmap.org/portal/). The gene perturbation effect (the Chronos dependency score) was calculated based on data from a cell depletion assay. A lower Chronos score indicates a higher likelihood that the gene of interest is essential for survival in a given cell line. A score of “0” indicates a gene that is not essential; correspondingly, “-1” is comparable to the median of all pan-essential genes (red line) (https://depmap.org/portal/). The prognosis of patients with ER^+^ tamoxifen/endocrine therapy-treated breast cancer stratified by *NUPR1* or *ERBB2* expression levels (low and high) were evaluated using Kaplan-Meier analysis from an extensive publicly available clinical breast cancer microarray database and web tool, Kaplan Meier plotter (https://kmplot.com/analysis/) 22. The sensitivity of MCF7 breast cancer cells to various chemotherapeutic agents was examined through analysis using a database (containing information on the sensitivity of 970 cell lines to 403 compounds) available on Genomics of Drug Sensitivity in Cancer (https://www.cancerrxgene.org/).

### Statistical analysis

Each experiment was repeated thrice. Data are presented as mean ± SEM. The significance of the difference was evaluated using one-way ANOVA. A “*”, “**” and “***” in the figures denotes a statistical significance with *p*-value < 0.05, < 0.01, and < 0.001, respectively, between the testing groups. In contrast, an “N.S.” denotes statistical insignificance between the testing groups.

## Results

### The endocrine therapy-resistant MCF7-TamC3 cells exhibit increased expression of NUPR1

**Figure 1:**
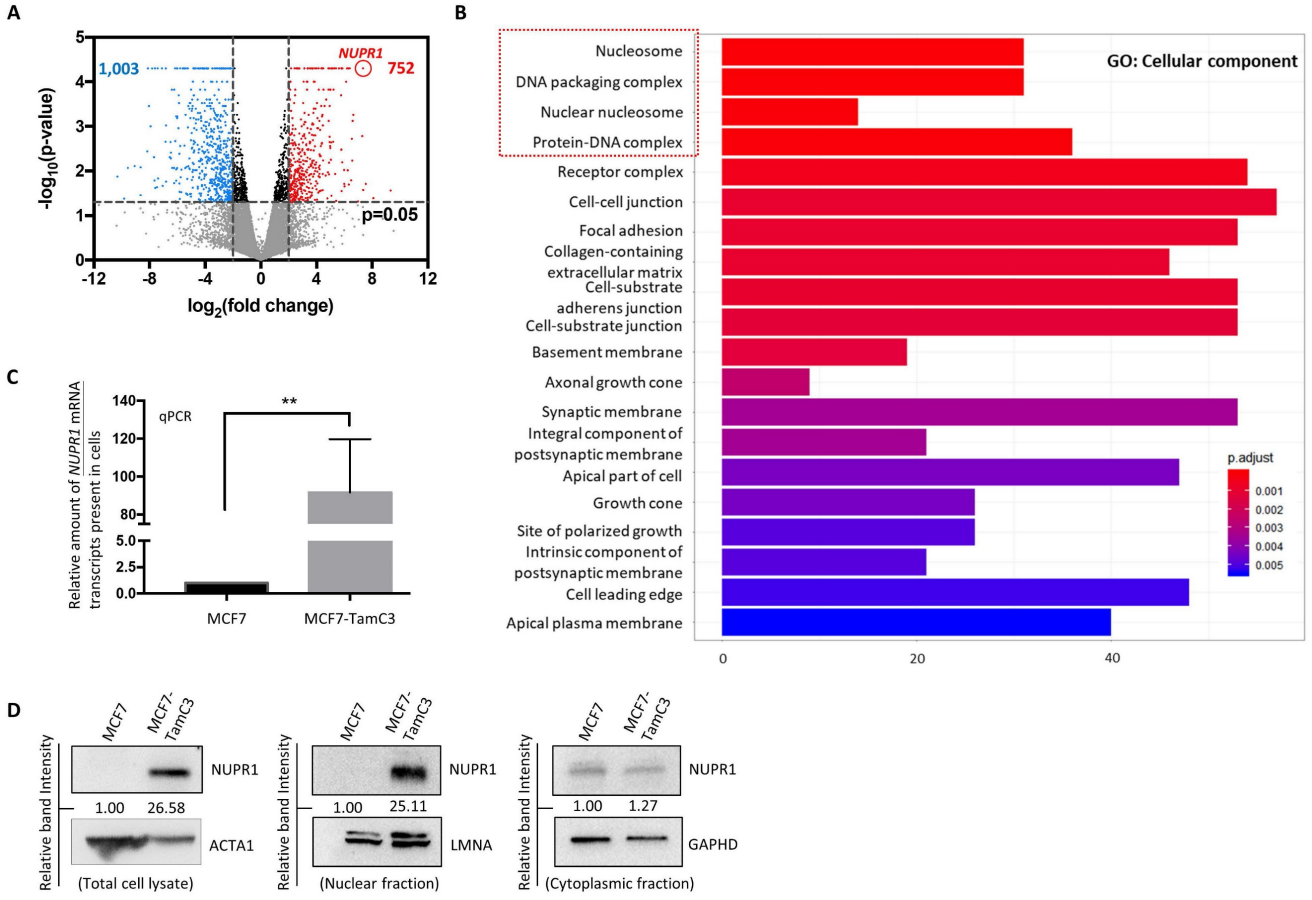
** MCF7-TamC3 cells exhibit increased expression of NUPR1. (A)** The expression levels of 58,219 genes were compared between the MCF7 and MCF7-TamC3 cells. Differentially expressed genes (DEGs) were selected with thresholds of fold change > 2 and *p*-value <0.05. Genes being upregulated (752, marked in red) and downregulated (1003, marked in blue) in MCF7-TamC3 cells are shown in the Volcano plot. **(B)** Histogram of Gene Ontology (GO) enrichment analysis of DEGs shows the top 20 differentially regulated cellular components in MCF7-TamC3 cells, compared to MCF7 cells. **(C and D)** The amount of the *NUPR1* mRNA transcripts and NUPR1 (total, nuclear, and cytoplasmic) protein presence in cells was determined by the qRT-PCR and western blot analysis, respectively. A “**” denotes a statistical significance of *p*-value <0.01 between the testing groups.

To identify potential molecules that play a role in the induction of endocrine therapy resistance, the transcriptome of MCF7 and the MCF7-derived endocrine therapy-resistant (estrogen-independent and Tamoxifen-resistant) MCF7-TamC3 breast cancer cells was analyzed using mRNAseq. A total of 1,755 genes were differentially expressed [*i.e.,* differentially expressed genes (DEGs)] in MCF7-TamC3, as compared to MCF7 cells (**Fig. 1A**). The gene ontology (GO) analysis showed that the DEGs were significantly enriched with functions on the structure/formation of chromosomes (*e.g.,* nucleosome, DNA packaging complex, nuclear nucleosome, and protein-DNA complex) (**Fig. 1B**). Among the 1,755 DEGs, *NUPR1* was the most upregulated gene in MCF7-TamC3 cells. NUPR1 is a transcription regulator primarily located in the nucleus of cells 23, 24. To confirm the results of the mRNAseq analysis, the qPCR and western blot analysis were performed to examine the expression levels of NUPR1 in cells, and the results showed that the *NUPR1* mRNA, total cellular NUPR1 protein and the nucleic NUPR1 protein expression levels are significantly upregulated in MCF7-TamC3 compared to MCF7 cells (**Fig. 1C and 1D**).

### Upregulation of NUPR1 in breast cancer patients with higher clinical stages and poorer prognostic outcomes

**Figure 2:**
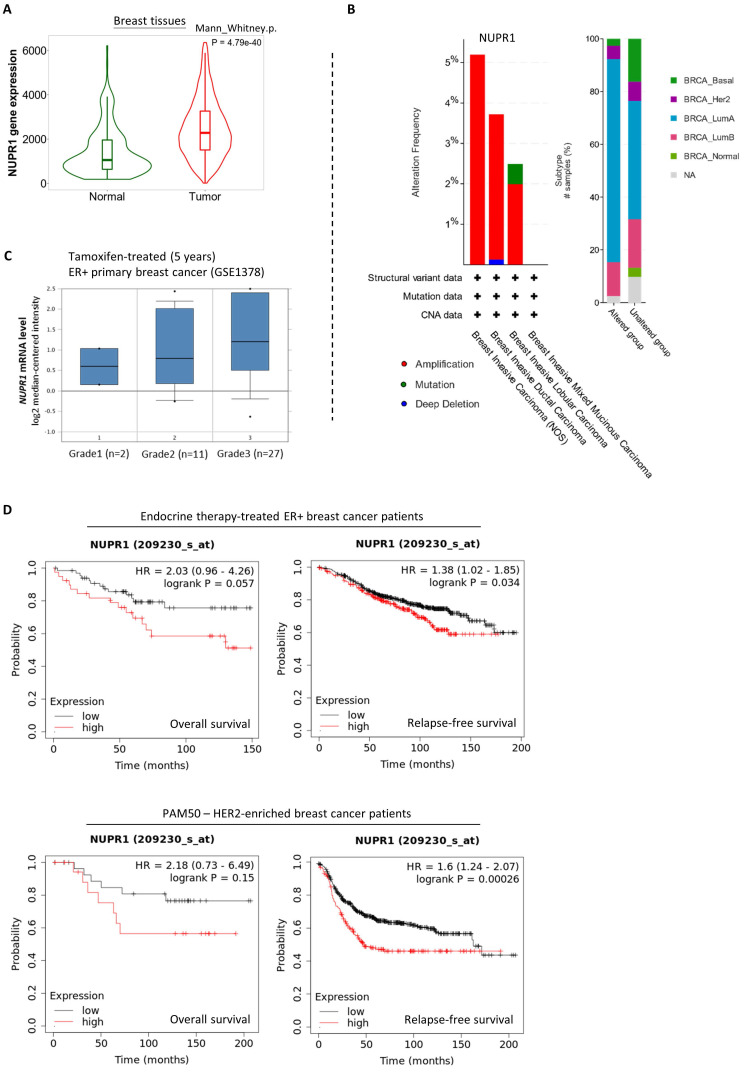
** Upregulation of NUPR1 in breast cancer patients with higher clinical stages and poorer prognostic outcomes**. **(A)** A violin plot shows the expression levels of the *NUPR1* gene in patients with non-paired normal (N=242) and breast tumor (N=7569) tissues in patients (TNMplot, www.tnmplot.com). **(B)** Analysis results show the alteration frequency of the *NUPR1* gene in 1080 breast cancer patients/samples of different subtypes (TCGA, PanCancer Atlas) (cBioPortal, www.cbioportal.org). CNA stands for “copy number alteration”. **(C)** Analysis of the expression level of *NUPR1* mRNA in Tamoxifen-treated (5 years) ER^+^ primary breast cancer (GSE1378) of different clinical grades (Oncomine, www.oncomine.org). **(D)** Kaplan-Meier survival (the overall and the relapse-free survival period) estimates of high (red line) or low (black line) *NUPR1* expression in the endocrine therapy-treated ER^+^ breast cancer patients and the ERBB2 (HER2)-enriched breast cancer patients (Kaplan-Meier plotter, https://kmplot.com).

Next, we sought to determine if NUPR1 upregulation readily promotes endocrine therapy resistance in ER^+^ breast cancer. Here, analysis using the online database and software available (TNMplot, www.tnmplot.com) revealed that the amount of *NUPR1* mRNA transcripts present in the tumor tissues is significantly higher than that in the normal tissues of breast cancer patients (**Fig. 2A**). Further analysis using data from cBioPortal (www.cbioportal.org) revealed that *NUPR1* gene amplification is present in approximately 2-5% of patients with breast invasive (lobular and ductal) carcinoma (**Fig. 2B, left panel**). Intriguingly, most of these samples (~40%) were obtained from patients with luminal A-subtype breast cancer (**Fig. 2B, right panel**). Expression of *NUPR1* mRNA was also higher in tamoxifen-treated ER^+^ primary breast tumors of higher clinical grades (Oncomine, www.oncomine.org) (**Fig. 2C**). As shown in **figure 2D**, Kaplan-Meier analysis (Kaplan-Meier plotter, https://kmplot.com) of expression cohorts of breast tumor showed that high *NUPR1* expression levels correlate with poor overall survival (despite not reaching statistical significance) and poor relapse-free survival in endocrine therapy-treated ER^+^ breast cancer patients. Intriguingly, high *NUPR1* expression levels also correlate with poor overall survival (despite not reaching statistical significance) and poor relapse-free survival in ERBB2-enriched breast cancer patients.

### NUPR1 plays an important role in maintaining the survival of ER^+^ breast cancer cells under estrogen deprivation

**Figure 3:**
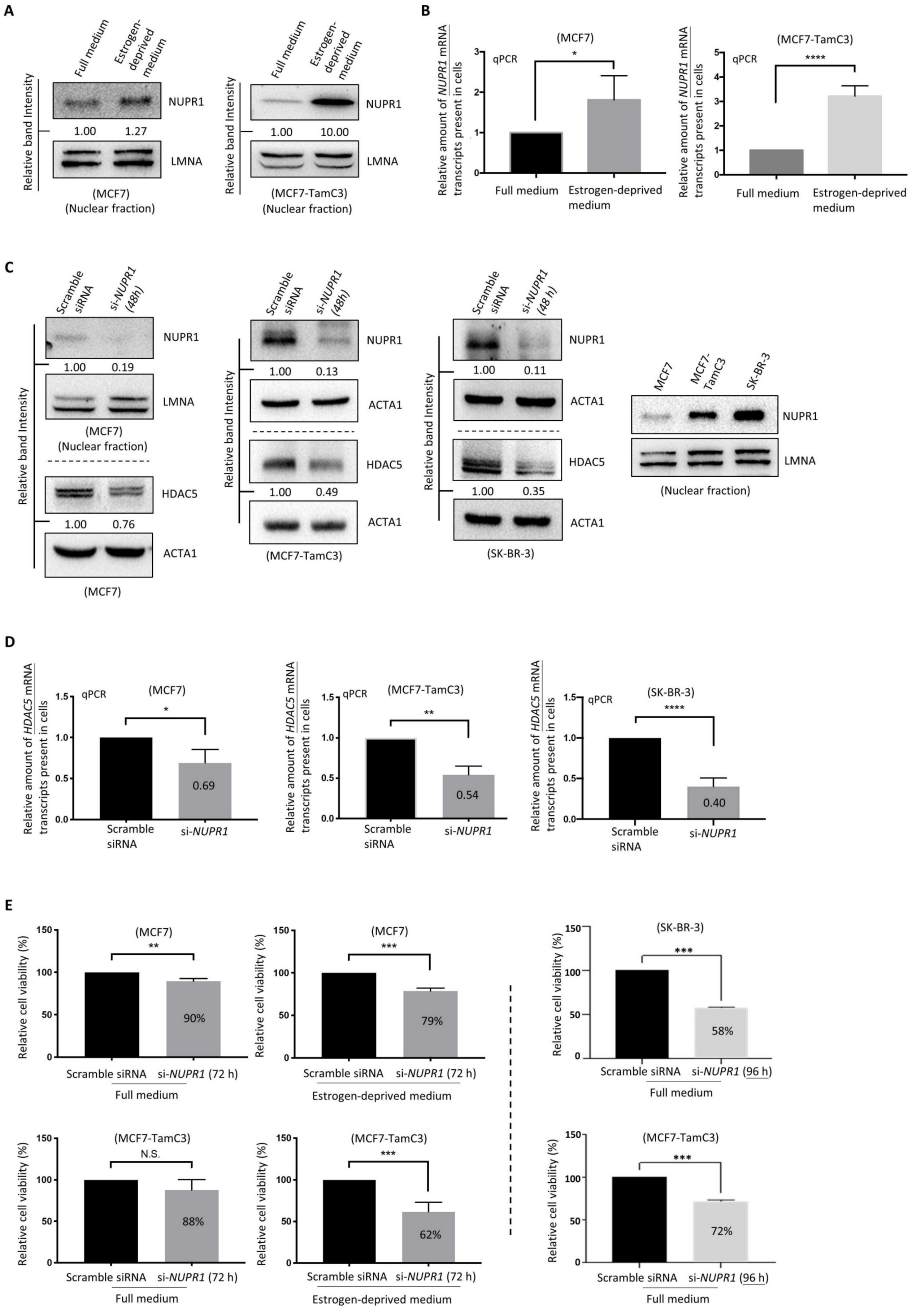
** NUPR1 plays an important role in the survival of ER+ breast cancer cells under estrogen-deprived conditions**. **(A and B)** MCF7 and MCF7-TamC3 cells were cultured under estrogen-contained (full medium) and estrogen-deprived conditions for 48 h. The expression level of *NUPR1* mRNA and the nucleic NUPR1 protein was examined by qRT-PCR and the western blot analysis, respectively. **(C and D)** MCF7-TamC3, SK-BR-3, and MCF7 cells were transfected with scramble or *NUPR1* siRNA for 48 h. The expression of HDAC5 protein and *HDAC5* mRNA transcripts in cells was determined by the western blot and qPCR analysis, respectively. **(E)** MCF7, MCF7-TamC3, and SK-BR-3 cells were transfected with *NUPR1* siRNA (si-*NUPR1*) and cultured under estrogen-containing and estrogen-depleted conditions for 72-96 h. The viability of cells was assessed using the MTT assay. A “*”, “**”, “***”, and “****” denotes a statistical significance of *p*-value <0.05, <0.01, <0.001, and <0.0001, respectively, between the testing groups.

Since the upregulation of *NUPR1* correlates with poor clinical outcomes in ER^+^ and ERBB2 (HER2)-enriched breast cancer patients, we sought to further confirm the role of NUPR1 in the induction of estrogen independence in ER^+^ breast cancer cells. Although MCF7 cells express less NUPR1 than MCF7-TamC3 cells under normal culturing (*i.e.*, estrogen-containing) conditions, both MCF7 and MCF7-TamC3 cells upregulated the expression of NUPR1 in response to estrogen deprivation (*i.e.*, aromatase inhibition-mimicking condition) (**Fig. 3A and 3B**). Of note, the estrogen-independent MCF7-TamC3 cells exhibited a higher response, in terms of NUPR1 upregulation, to estrogen deprivation compared to MCF7 cells.

We previously reported that HDAC5 (an epigenetic regulator) upregulation promotes estrogen independence and Tamoxifen resistance in ER^+^ breast cancer cells 15; however, the expression regulation of HDAC5 was unclear. Interestingly, downregulation of *NUPR1* by siRNA (*i.e.*, si-*NUPR1* treatment) decreased the expression of HDAC5 in MCF7-TamC3 cells (**Fig. 3C and 3D**). Despite the low endogenous NUPR1 expression level, downregulation of *NUPR1* (using the nucleic NUPR1 protein as a marker) by siRNA still decreased the expression of NUPR1 in MCF7 cells. We noticed that the ERBB2 (HER2)-enriched subtype-like [ER^-^, ERBB2 (HER2)^+/high^] SK-BR-3 breast cancer cells express a large amount of the nucleic NUPR1 protein endogenously, as compared to MCF7 and MCF7-TamC3 cells, and the si-*NUPR1* treatment also decreased the expression of HDAC5 in SK-BR-3 cells (**Fig. 3C and 3D**).

At the cellular level, the downregulation of *NUPR1* by siRNA hampered the cell viability further in MCF7 and MCF7-TamC3 cells cultured under the estrogen-deprived condition (79% and 62%, respectively, 72 h post-treatment), as compared to cells cultured under the normal condition (90% and 88%, respectively, 72 h post-treatment) (**Fig. 3E**). It is also worth noting that although *NUPR1* siRNA exerted limited cell viability effects in MCF7-TamC3 cells under normal condition 72 h post-treatment, prolonged *NURP1* siRNA treatment (96 h) significantly reduced the viability of MCF7-TamC3 cells (72%) (**Fig. 3E**). Here, *NUPR1* siRNA treatment also decreased the viability of the NUPR1 highly expressing SK-BR-3 breast cancer cells (**Fig. 3E**). These results suggest that NUPR1 plays an essential role in maintaining the survival of ER^+^ breast cancer cells, especially under the estrogen-deprived condition.

### NUPR1 positively regulates the expression of ERBB2 in breast cancer cells

**Figure 4:**
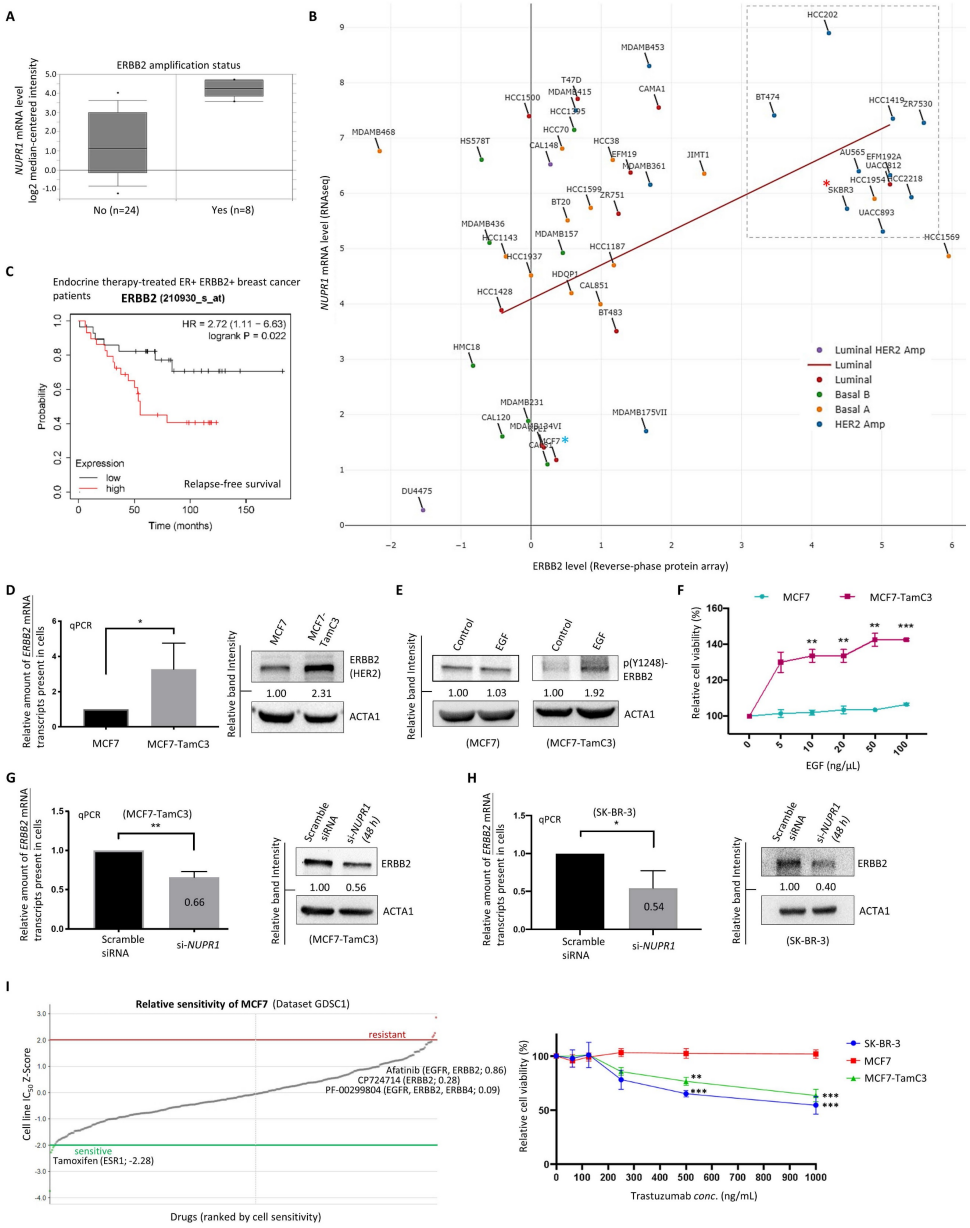
** The ER^+^ MCF7-TamC3 cells exhibit molecular and cellular properties of the ERBB (HER2)-enriched breast cancer cells**. **(A)** Analysis of the expression level of *NUPR1* mRNA in breast cancer tissue with ERBB2 (HER2) amplification (N=8) and without ERBB2 amplification (N=24) (GSE10087) (Oncomine, www.oncomine.com/). **(B)** Analysis of the expression correlation between the ERBB2 (HER2) protein and *NUPR1* gene in human breast cancer cells of different molecular subtypes (DepMap, www.depmap.org/portal/). **(C)** Kaplan-Meier survival (the relapse-free survival period) estimates of high (red line) or low (black line) *ERBB2* expression in endocrine therapy-treated ER^+^ ERBB2^+^ breast cancer patients (Kaplan-Meier plotter, https://kmplot.com). **(D)** The endogenous expression level of *ERBB2* mRNA and ERBB2 protein was determined by qRT-PCR and the western blot analysis, respectively. **(E)** MCF7 and MCF7-TamC3 cells were treated with and without 100 ng/mL epidermal growth factor (EGF) for 24 h under serum-free conditions. The expression of p(Y1248)-ERBB2 (phosphorylated ERBB2) was examined by western blotting. **(F)** MCF7 cells and MCF7-TamC3 cells were incubated with indicated concentrations of EGF for 72 h under serum-free conditions, after which cell viability was assessed by the MTT assay. **(G and H)** Cells were treated with *NUPR1* siRNA (si-*NUPR1*) for 48 h. The expression level of *ERBB2* mRNA and ERBB2 protein was examined by qRT-PCR and the western blot analysis, respectively. **(I)** (Left) Bioinformatic analysis results showing the sensitivity of MCF7 cells to various chemotherapeutic agents (Genomics of Drug Sensitivity in Cancer, https://www.cancerrxgene.org/). (Right) Breast cancer cells were treated with Trastuzumab for 72 h. The cell viability was assessed using the MTT assay. A “*”, “**”, and “***” denotes a statistical significance of *p*-value <0.05, <0.01, and <0.001, respectively, between the testing groups.

We previously demonstrated that HDAC5 negatively regulates the expression of the microRNA molecule, *miR-125a-5p*, and the overexpression of *miR-125a-5p* decreases the expression of ERBB2 in ER^+^ breast cancer cells 15. Thus, we examined possible relationships between the expression of NUPR1 and ERBB2 in breast cancer cells. Analysis using the database available online (Oncomine, www.oncomine.com/) revealed that the amount of *NUPR1* mRNA transcripts present in the ERBB2-amplified breast cancer tissues is higher than those breast cancer tissues without ERBB2-amplification (**Fig. 4A**). A positive correlation between the expression of *NUPR1* mRNA and ERBB2 (HER2) protein is also found exist in luminal-like breast cancer cell lines (Pearson correlation coefficient, ρ = 0.362) (DepMap, https://depmap.org/portal/) (**Fig. 4B**). Notably, most breast cancer cell lines with ERBB2/HER2 amplification, including the ERBB2 (HER2)-enriched subtype-like SK-BR-3 (labelled with a red colored asterisk in the figure), exhibit high levels of *NUPR1* mRNA expression (**Fig. 4B**). In contrast, the expression levels of *NUPR1* mRNA and ERBB2 protein in the luminal-A subtype-like MCF7 (labelled with a blue colored asterisk in the figure) breast cancer cells are low, as expected (**Fig. 4B**). Prognostically, Kaplan-Meier analysis (Kaplan-Meier plotter, https://kmplot.com) of expression cohorts of breast tumor showed that high *ERBB2* mRNA expression levels correlate with poor relapse-free survival in endocrine therapy-treated, ER^+^ ERBB2^+^, breast cancer patients (**Fig. 4C**).

Subtype switch between primary and recurrent/metastatic breast tumors has frequently been reported 3, 9. Intriguingly, the estrogen-independent and Tamoxifen-resistant MCF7-TamC3 cells not only exhibited increased expression of ERBB2 (**Fig. 4D**) but also showed significant increased sensitivity to epidermal growth factor (EGF) compared to MCF7 cells. The western blot and MTT cell viability analysis results showed that EGF treatment induced ERBB2 phosphorylation and significantly increased the viability of the ERBB2 highly-expressed MCF7-TamC3 cells. In contrast, its effects on ERBB2 phosphorylation and the viability of the ER^+^ ERBB2^-/low^ MCF7 cells were limited (**Fig. 4E and 4F**). Noticeably, EGF treatment promoted the migration of MCF7-TamC3 but not MCF7 cells *in vitro* (**Fig. S1**). Downregulation of *NUPR1* by siRNA decreased the amount of both the *ERBB2* mRNA transcripts and the ERBB2 proteins present in the ER^+^ ERBB2^high^ MCF7-TamC3 and the ER^-^ ERBB2^high^ (ERBB2-enriched subtype-like) SK-BR-3 breast cancer cells (**Fig. 4G and 4H**).

MCF7 cells are susceptible to the estrogen-ER signaling pathway-targeting agents like tamoxifen but resistant (relatively insensitive) to the EGFR/ERBB2 signaling-pathway inhibitors, including Afatinib and CP724714 (Genomics of Drug Sensitivity in Cancer, https://www.cancerrxgene.org/) (**Fig. 4I, left panel**). Herceptin is an ERBB2-specific monoclonal antibody widely used clinically for treating patients with ERBB2-enriched breast cancer. Here, the MTT assay results showed that Herceptin reduced the viability of the ER^-^ ERBB2^high^ SK-BR-3 cells but not of the ER^+^ ERBB2^-/low^ MCF7 cells, as expected (**Fig. 4I, right panel**). Interestingly, Herceptin reduced the viability of the ER^+^ ERBB2^high^ MCF7-TamC3 cells in a concentration-dependent manner, indicating that MCF7-TamC3 cells are not only hyper-sensitive to EGF but also exhibit increased reliance on the EGF-ERBB2-signaling pathway for cell survival, comparing to MCF7 cells (**Fig. 4I**). Taken together, these results indicate that NUPR1 positively regulates the expression of ERBB2 and promotes the EGF-ERBB2 pathway-related survival in breast cancer cells. The upregulation of NUPR1 may increase cell sensitivity to and the dependence of EGF for viability, helping to maintain the survival of MCF7-TamC3 cells under estrogen-deprived conditions.

### NUPR1 regulates the TP53-signaling pathway and BIRC5 expression in breast cancer cells

**Figure 5:**
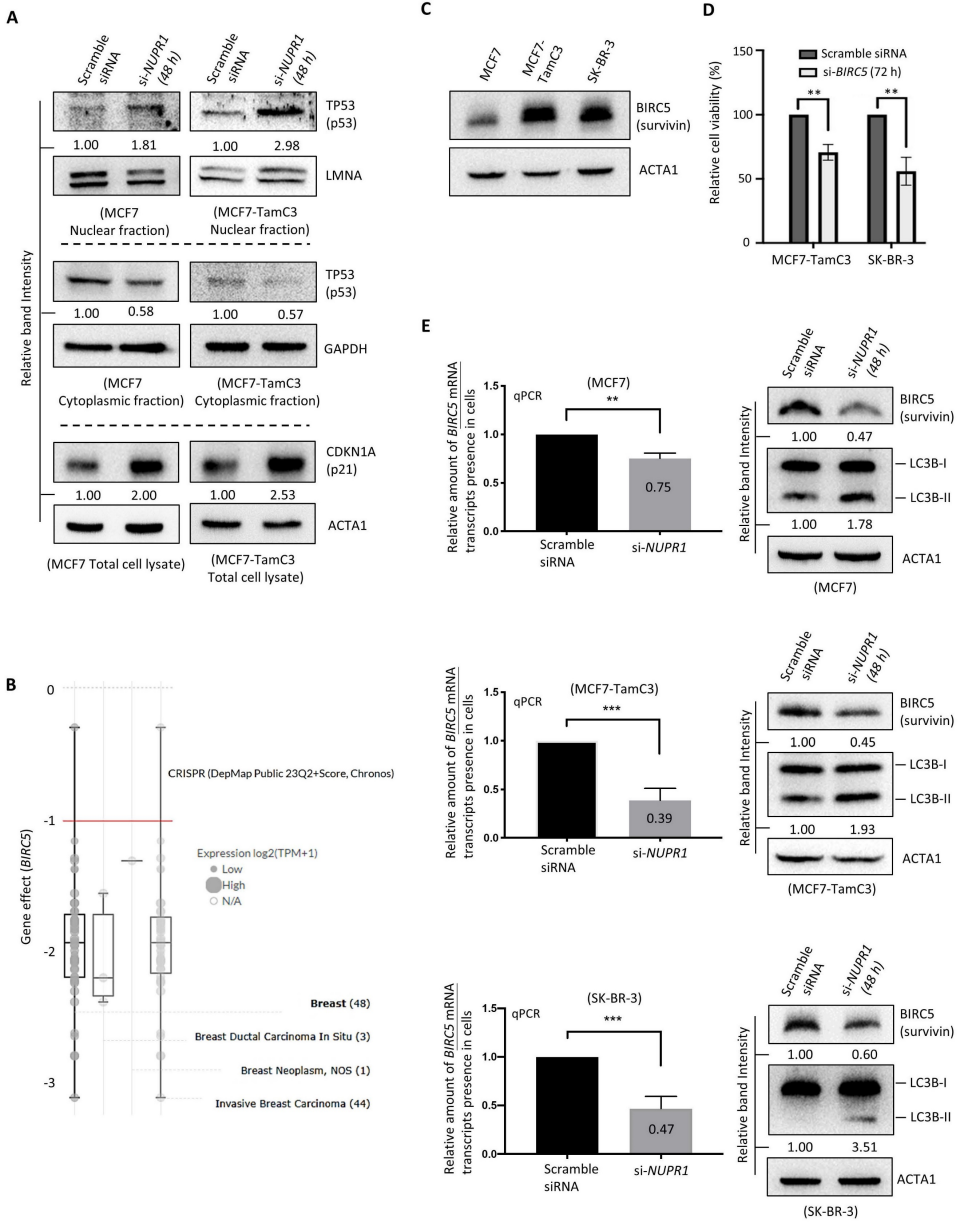
** NUPR1 regulates the expression of BIRC5 in breast cancer cells. (A)** Cells were transfected with scramble or *NUPR1* siRNA (si-*NUPR1*) for 48 h. The expression of different proteins was determined by the western blot analysis. **(B)** A graph showing the expression level and gene perturbation effect (the Chronos dependency score) of *BIRC5* in various types of breast cancer cells was generated using software and an online database (DepMap, https://depmap.org/portal/). A lower Chronos score indicates a higher likelihood that the gene of interest is essential for survival in a given cell line. A score of “0” indicates a gene that is not essential; correspondingly, “-1” is comparable to the median of all pan-essential genes (red line). **(C)** The endogenous expression level of BIRC5 in MCF7, MCF7-TamC3, and SK-BR-3 cells was determined by the western blot analysis. **(D)** Cells were transfected with scramble or *BIRC5* siRNA (si-*BIRC5*) for 72 h. The viability of cells was examined by the MTT assay. **(E)** Cells were transfected with scramble or *NUPR1* siRNA (si-*NUPR1*) for 48 h. The expression of *BIRC5* mRNA transcripts and BIRC5 protein and the conversion of LC3B-II were determined by qRT-PCR and the western blot analysis, respectively. A “**” and “***” denotes a statistical significance of *p*-value <0.01 and <0.001, respectively, between the testing groups.

We previously reported that MCF7-TamC3 cells exhibit decreased expression of TP53 (p53) and increased expression of a known anti-apoptotic and pro-mitotic molecule, BIRC5 (Survivin) (**Table 1**) 25, 26, as compared to MCF7 cells 15. However, the effect of NUPR1 on TP53 is controversial, as different studies showed differential regulatory roles of NUPR1 on TP53 27, 28. Thus, we sought to determine if NUPR1 regulates the expression of TP53 and its downstream transcription target, *BIRC5*, in breast cancer cells. Here, downregulation of *NUPR1* by siRNA increased the expression of the nucleic TP53 and concurrently decreased the expression of cytoplasmic TP53 in MCF7-TamC3 and MCF7 cells (**Fig. 5A**). In aligned with the function of TP53 as a positive protein expression-regulator of CDKN1A (p21) 27, 29, the expression of CDKN1A was increased in cells with *NUPR1* downregulations (**Fig. 5A**).

BIRC5 plays an important role in the survival of various types of breast cancer cells [*i.e.*, a Chronos Dependency Score (gene effect) of less than -1] (DepMap, https://depmap.org/portal/) (**Fig. 5B**). TP53 is a negative transcription-regulator of the *BIRC5* gene 30, 31. In contrast, ERBB2 and its ligand, EGF, positively regulate the expression of BIRC5 in cells 32, 33. Both the ER^+^ MCF7-TamC3 and the ERBB2^+/High^ SK-BR-3 cells express a large amount of BIRC5, as compared to MCF7 cells (**Fig. 5C**). Downregulation of *BIRC5* by siRNA decreased the viability of MCF7, MCF7-TamC3, and SK-BR-3 cells, highlighting the critical role of BIRC5 in the survival of the examined breast cancer cells (**Fig. 5D**). The qPCR and western blot analysis results showed that *NUPR1* downregulation by siRNA decreased the amount of the *BIRC5* mRNA transcripts and the BIRC5 proteins present in MCF7 and MCF7-TamC3 cells (**Fig. 5E**). Like the results of MCF7 and MCF7-TamC3 cells with *NUPR1* downregulations, the same treatment decreased the amount of the *BIRC5* mRNA transcripts and the BIRC5 proteins present in SK-BR-3 cells (**Fig. 5E**). In align with the role of BIRC5 as an autophagy suppressor, *NUPR* siRNA treatment increased the conversion of LC3B-II, which is a molecular marker for autophagy induction (**Fig. 5E**) 34, 35. Collectively, these results suggest that the upregulation of NUPR1 partially promotes the survival of MCF7-TamC3 breast cancer cells by switching the survival reliance from the estrogen-ER-signaling to the EGF-ERBB2-signaling, modulating the TP53-signaling pathway, and upregulating the expression of BIRC5.

## Discussion

Emerging evidence shows that molecular subtype conversion frequently occurs during the development of metastatic or drug-resistant breast cancer 3-5. For instance, in a cohort of 57 patients with matched breast cancer and lung or pleural metastasis (BCLM), Klebe *et al*. reported that out of 28 patients initially diagnosed as luminal A breast tumor (PAM50), 10 recurred as luminal B and four recurred as ERBB2-enriched lung metastasis 3. It has also been reported that the percentage of different tumor cell sub-populations within breast tumors of patients can be changed dramatically during aromatase inhibitor treatment (AI, endocrine therapy) 7. However, the etiology of such molecular subtype conversion between primary breast tumors and metastatic lesions is still unclear. In the current study, we found that the ER^+^ luminal A-like MCF7 breast cancer cells-derived ER^+^, estrogen-independent, and Tamoxifen-resistant MCF7-TamC3 cells exhibit increased expression of NUPR1 and molecular/cellular characteristics, which resemble those of the EGF-dependent ERBB2-enriched subtype-like breast cancer cells.

NUPR1 is an intrinsically disordered protein (IDP) that has a nuclear localization sequence (NLS) to allow for nuclear translocation 36, 37. It is a stress-inducible protein involved in gene transcription, partly through DNA binding 36, 38. However, the role of NUPR1 on cell survival remains controversial. It has been demonstrated that NUPR1 binds to poly [ADP-ribose] polymerase 1 (PARP1) and inhibits the activity of PARP1 in cancer cells 23. Genetic inhibition of NUPR1 induces PARP1 over-activation and decreases ATP production and cell viability in cancer cells 23. Pharmacologically, inhibiting NUPR1 by ZZW-115 increases the mitochondrial and cellular ROS production in pancreatic cancer cells and induces cancer cell death 23. In contrast, NUPR1 plays an important role in palmitate-induced apoptosis in human articular chondrocytes 39. Furthermore, it has been demonstrated that quercetin upregulates NUPR1 expression and induces ROS-NUPR1-dependent autophagic cell death in osteosarcoma cells 40.

Dysregulation (overexpression) of the epigenetic regulator, HDAC5, is known to promote endocrine therapy resistance in breast cancer 15, 41. We previously demonstrated that the downregulation of HDAC5 partially restores the sensitivity to Tamoxifen and estrogen deprivation in MCF7-TamC3 cells 15. Xue *et al*. further showed that HDAC5-mediated deacetylation and nuclear localization of SOX9 are critical for maintaining Tamoxifen resistance in breast cancer 41. In the current study, we found that MCF-TamC3 cells exhibit epigenetic dysregulation-like molecular characteristics, as revealed by the results of the mRNAseq and the GO analysis. At the molecular level, we discovered NUPR1 as a positive expression regulator of HDAC5 in breast cancer cells. We also found NUPR1 as a negative expression regulator of the nuclear TP53 in cancer cells. Ring finger protein 1B (RING1B) is an E3 ubiquitin ligase crucial for the monoubiquitination of histone H2A (H2AK119ub1). RING1B can also control gene expression and compact chromatin *via* an H2A ubiquitin ligase activity-independent mechanism 42. Although the exact regulatory effect on RING1B is unclear, it has been demonstrated that NUPR1 binds to the C-terminal region of the RING1B protein of the Polycomb complex 43. Interestingly, NUPR1 forms a protein complex with a histone citrullination enzyme, peptidyl arginine deiminase 4 (PADI4/PAD4), in cancer cells 44, and PADI4 interacts with the C-terminus of TP53 and functions as a TP53 co-repressor 45, 46. Despite the effect of “NUPR1-binding” on PADI4 is still unclear, it has been shown that inhibition of PADI4 upregulates the expression of different TP53 target genes like *CDKN1A* (*p21*) and induces apoptosis of cells 45. Thus, upregulation of NUPR1 might promote the induction of endocrine therapy resistance (estrogen independence and Tamoxifen resistance) in ER^+^ breast cancer cells through modulations on multiple cell surface receptor-related/unrelated signaling pathways and molecules like BIRC5 and ERBB2, shifting the cellular properties towards other breast cancer subtypes like ERBB2 (HER2)-enriched breast cancer.

Autophagy is a double-edged sword, as upregulation of this process has been shown to promote both the survival and death of cells. The role of autophagy in the induction of endocrine therapy resistance is controversial. It has been demonstrated that Tamoxifen upregulates autophagy and induces autophagy-related cell death in MCF7, U87 (glioblastoma), ARPE-19 (retinal pigment epithelia), and 661W (photoreceptor) cells 47-49. We previously showed that the endocrine therapy-resistant (estrogen-independent and Tamoxifen-resistant) MCF7-TamC3 cells exhibit lowered baseline autophagic flux levels [decreased expression of ATG12-ATG5 conjugate, increased expression of SQSTM1 (p62), and reduced rate of degradation of the SQSTM1 protein] compared to MCF7 breast cancer cells 15. We also showed that Tamoxifen downregulates the expression of BIRC5 and increases the conversion of LC3B-II in MCF7 and ZR-75-1 (ER^+^ breast cancer) cells 15. Despite BIRC5 being widely known as an antiapoptotic molecule that inhibits the activity of various caspases and a pro-mitotic molecule that is crucial for the formation of the chromosomal passenger complex (CPC) during mitosis, we recently discovered a novel role of BIRC5 on autophagy regulation 34, 35. It physically interacts with ATG12-ATG5 conjugate, ATG12, and ATG5, subsequently inhibiting the formation of the ATG12-ATG5-ATG16L protein complex, which is required for the autophagophore elongation and maturation in cells 34. Notably, Wang *et al.* demonstrated that Tamoxifen induces NUPR1 expression, and NUPR1 maintains Tamoxifen resistance in the ER^+^ MCF-7TamR breast cancer cells 50. They also demonstrated that NUPR1 directly suppresses *BECN1* (a molecule important for the initiation of autophagy) transcription in MCF-7TamR cells 50. Thus, a lowered baseline autophagy level may favor the survival of the ER^+^ endocrine therapy-resistant MCF7-TamC3 cells under estrogen-deprived conditions and the Tamoxifen treatment, as excessive autophagy may lead to cell death.

## Conclusion

**Figure 6:**
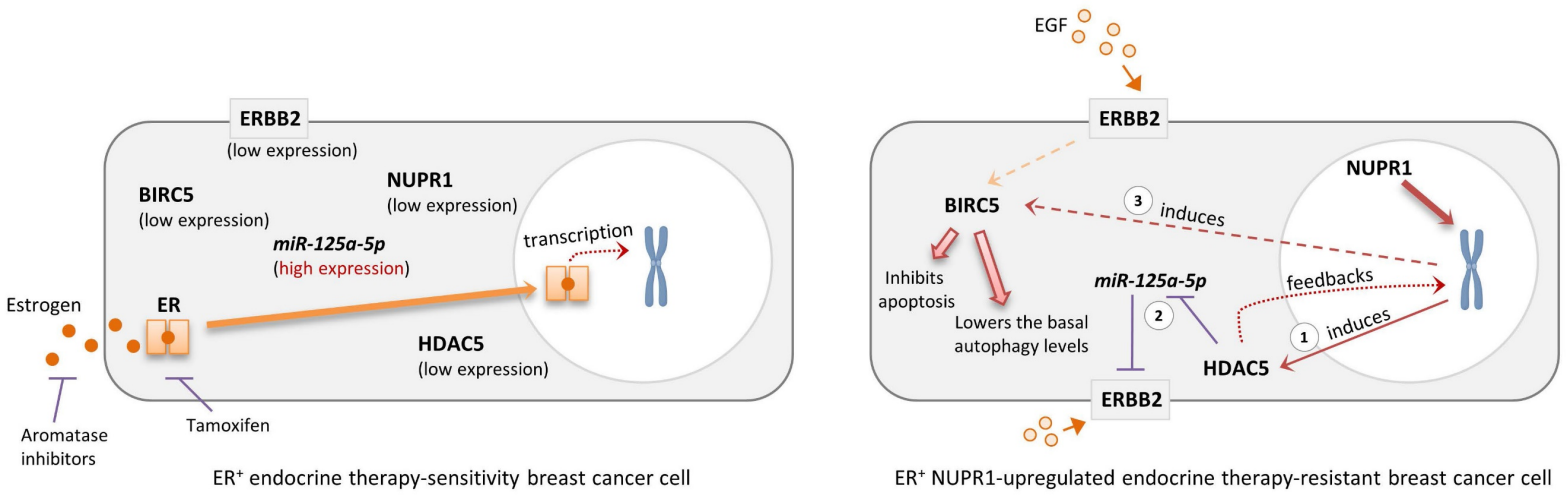
** Schematic diagram showing the possible role of NUPR1 in regulating the expression of HDAC5, BIRC5, and ERBB2 in the ER^+^ endocrine therapy-resistant breast cancer cells**. NUPR1 positively regulates the mRNA transcription and protein expression of the epigenetic regulator, HDAC5 (step 1), in ER^+^ breast cancer cells. The upregulation of HDAC5 suppresses the expression of *miR-125a-5p* (a negative regulator of ERBB2), leading to the increased expression of ERBB2 (step 2). HDAC5 and ERBB2 upregulation promotes the expression of BIRC5, subsequently inhibiting the activation of apoptosis and lowering the basal autophagic flux levels in the ER^+^ endocrine therapy-resistant breast cancer cells (step 3).

Upregulation of NUPR1 can be found in the ER^+^, estrogen-independent, tamoxifen-resistant breast cancer cells and high mRNA expression of *NUPR1* correlates with poor clinical outcomes in ER^+^ endocrine therapy-treated breast cancer patients. Because NUPR1 positively regulates the expression of HDAC5, ERBB2, and BIRC5 in both the ER^+^ luminal A subtype-like and the ERBB2-enrich subtype-like breast cancer cells, targeting NUPR1 or their downstream regulating molecules like HDAC5 and BIRC5 may offer a potential strategy for overcoming resistance to endocrine therapy in patients with ER^+^ breast cancer (**Fig. 6**). Additionally, targeting ERBB2 (such as the use of Trastuzumab) could be an effective treatment for patients originally diagnosed with luminal A breast cancer with subsequently found to exhibit luminal B-ERBB2^+^ characteristics and resistance to endocrine therapy after prolonged treatment. Trastuzumab Deruxtecan, an antibody-drug conjugate (ADC) consisting of a humanized anti-ERBB2 (HER2) monoclonal antibody linked to the topoisomerase I inhibitor Deruxtecan, represents a novel therapeutic option for patients with advanced hormone receptor-positive (HR^+^, *e.g.*, ER^+^) breast cancer. A recent phase 3 trial demonstrated that the use of Trastuzumab Deruxtecan significantly improved survival outcomes in patients with HR^+^/ERBB2^Low^ breast cancer that was refractory to endocrine therapy compared to the control group 51. These findings further underscore the potential of the use of anti-ERBB2 therapy in patients with ER^+^ hormone-refractory (or endocrine therapy-resistant) breast cancer. Re-biopsy of tumor tissues to assess the real-time status of ERBB2 expression in endocrine therapy-resistant ER^+^ breast cancer may be necessary for the decision to use Trastuzumab Deruxtecan, particularly in cases of NUPR1-overexpressing breast tumors.

## Supplementary Material

Supplementary figure.

## Figures and Tables

**Table 1:** ** The fifteen nearest neighbors of *BIRC*5 based on single cell type RNA expression** (*i.e.*, similar genes in terms of expression profile; The Human Protein Atlas, https://www.proteinatlas.org/). Cluster 52 - cell proliferation, and Cluster 70 -Spermatid development.

Neighbor	Description	Spearman Correlation	Cluster ID (Function)
*AURKB*	Aurora kinase B	0.8403	52
*HMMR*	Hyaluronan mediated motility receptor	0.8342	52
*NUSAP1*	Nucleolar and spindle associated protein 1	0.8256	52
*CENPN*	Centromere protein N	0.8245	52
*SKA3*	Spindle and kinetochore associated complex subunit 3	0.8194	52
*UBE2T*	Ubiquitin conjugating enzyme E2 T	0.8091	52
*CENPA*	Centromere protein A	0.8065	52
*NDC80*	NDC80 kinetochore complex component	0.8027	52
*PRC1*	Protein regulator of cytokinesis 1	0.7927	52
*TROAP*	Trophinin associated protein	0.7904	52
*CCNA2*	Cyclin A2	0.7874	52
*KIF23*	Kinesin family member 23	0.7862	70
*PRR11*	Proline rich 11	0.7840	52
*CDC20*	Cell division cycle 20	0.7797	52
*ZWINT*	ZW10 interacting kinetochore protein	0.7795	52
