# Supplementary material for: NUPR1 contributes to endocrine therapy resistance by modulating BIRC5 expression and inducing luminal B-ERBB2+ subtype-like characteristics in estrogen receptor-positive breast cancer cells
Source: J Cancer. 2025 Feb 11;16(5):1694–708. doi: 10.7150/jca.105425 (PMC11843241; doi:10.7150/jca.105425)
Supplement: Supplementary file 1 — Supplementary figure. [file jcav16p1694s1.pdf]

Figure S1 – MCF7-TamC3 cells exhibit increased sensitivity to EGF. MCF7 and MCF7-TamC3 cells were treated with or without 100 ng/mL EGF in serum-free mediums. Cell migration was examined 24 h post-EGF stimulation. Cell migration was quantified by measuring the mean distance between the edges of the interval.

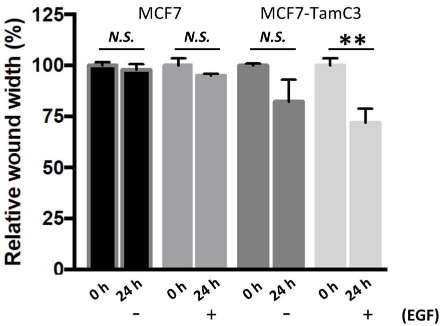
MCF7
MCF7-TamC3
Relative wound width (%)
125
100
75
50
25
0
N.S.
N.S.
N.S.
**
0 h
24 h
0 h
24 h
0 h
24 h
0 h
24 h
-
+
-
+
(EGF)

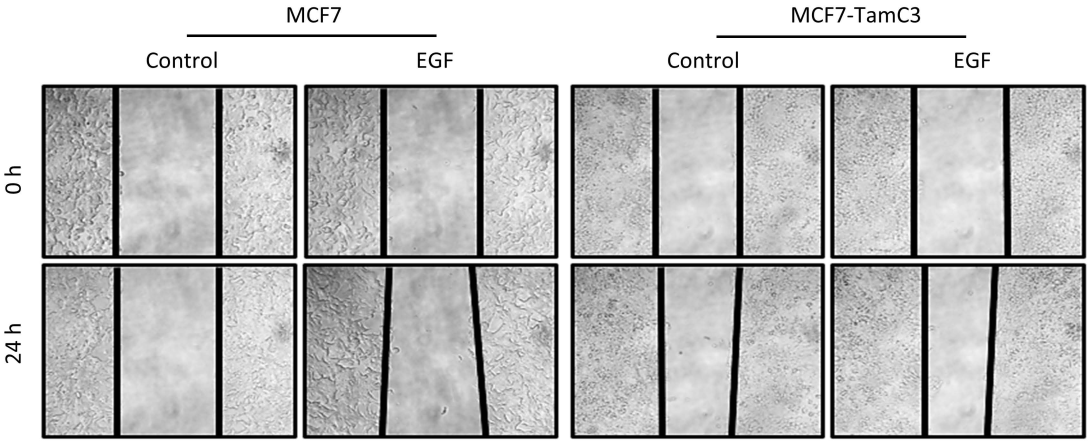
MCF7
MCF7-TamC3
Control
EGF
Control
EGF
0 h
24 h
